# Type XVII collagen: Relevance of distinct epitopes, complement-independent effects, and association with neurological disorders in pemphigoid disorders

**DOI:** 10.3389/fimmu.2022.948108

**Published:** 2022-08-10

**Authors:** Bianca Opelka, Enno Schmidt, Stephanie Goletz

**Affiliations:** ^1^ Lübeck Institute of Experimental Dermatology (LIED), University of Lübeck, Lübeck, Germany; ^2^ Department of Dermatology, University of Lübeck, Lübeck, Germany

**Keywords:** BP180, epitopes, pemphigoid, autoimmunity, bullous disease, autoantigen

## Abstract

Pemphigoid diseases (PD) are autoimmune skin blistering diseases characterized by autoantibodies directed against proteins of the cutaneous basement membrane zone (BMZ). One of the major antigens is type XVII collagen (BP180), a transmembrane glycoprotein, which is targeted in four PDs: bullous pemphigoid, mucous membrane pemphigoid, linear IgA dermatosis, and pemphigoid gestationis. To date, different epitopes on BP180 have been described to be recognized by PD disease patients’ autoantibodies. Different BP180 epitopes were associated with distinct clinical phenotypes while the underlying mechanisms are not yet fully understood. So far, the main effects of anti-BP180 reactivity are mediated by Fcγ-receptors on immune cells. More precisely, the autoantibody–antigen interaction leads to activation of complement at the BMZ and infiltration of immune cells into the upper dermis and, by the release of specific enzymes and reactive oxygen species, to the degradation of BP180 and other BMZ components, finally manifesting as blisters and erosions. On the other hand, inflammatory responses independent of Fcγ-receptors have also been reported, including the release of proinflammatory cytokines and internalization and depletion of BP180. Autoantibodies against BP180 can also be found in patients with neurological diseases. The assumption that the clinical expression of PD depends on epitope specificity in addition to target antigens, autoantibody isotypes, and antibody glycosylation is supported by the observation that epitopes of PD patients differ from those of PD patients. The aim of the present review is to describe the fine specificities of anti-BP180 autoantibodies in different PDs and highlight the associated clinical differences. Furthermore, the direct effects after binding of the autoantibodies to their target are summarized.

## Introduction

The skin is the largest human organ. It serves as a barrier and protects the body from environmental influences, including heat, cold, dehydration, UV radiation, and pathogens, as well as many toxic and immunogenic substances. The integrity of the skin is established by cell–cell contacts among keratinocytes and the adhesion of the epidermis to the dermis at the dermo-epidermal junction (DEJ). Proteins of these structures target antigens in various autoimmune bullous disorders, i.e., pemphigus and pemphigoid diseases (PD) (1, 2).

In addition to providing structural integrity, the DEJ has multiple functions and regulates epithelial–mesenchymal interaction during skin homeostasis, growth, and wound healing; serves as a permeability barrier; and participates in signal transduction (3–8). The individual layers of the DEJ can electron microscopically be subdivided in the lamina lucida, directly adjacent to the plasma membrane of basal keratinocytes, the lamina densa, and the sublamina densa. The DEJ is mainly composed of widely conserved molecules, including collagen type IV, laminins, nidogen, and perlecan (6). Within the DEJ-specialized cell-substratum adhesion sites, the so-called hemidesmosomes mediate the interaction between the cytoskeleton and, *via* anchoring filaments and anchoring fibrils, dermal collagens (5, 9). Hemidesmosomes of the epidermis and surface-close epithelia are composed of an intracellular plaque where the intermediated filaments keratins K5 and K14 link with the plectin isoform 1a (P1a) and the bullous pemphigoid antigen 1 isoform e (BPAG1e), also termed BP230. The latter two hemidesmosomal molecules interact with the two transmembrane molecules α6β4 integrin and BP180 (also called type XVII collagen or BPAG2). As the fifth hemidesmosomal protein, the transmembrane tetraspanin CD151 associates with the extracellular loop of α6 integrin. In the extracellular space, α6β4 integrin and BP180 connect with anchoring filaments, mainly consisting of laminin 332, laminin 331, and uncein. Anchoring filaments interact with anchoring fibrils, mainly consisting of type VII collagen, that finally links with dermal collagen (9–11).

In PD, autoantibodies are directed against proteins of the DEJ, primarily BP180, BP230, laminin 332, the p200 antigen (laminin γ1), and collagen type VII (12–15).

The present review focuses on BP180, the main autoantigen of the DEJ, and highlights the fine autoantibody specificities against this autoantigen targeted in four PD, i.e. bullous pemphigoid (BP), mucous membrane pemphigoid (MMP), linear IgA dermatosis, and pemphigoid gestationis (16, 17). In addition, this review summarizes the described direct effects immediately after binding of anti-BP180 antibodies to their cellular target.

## Epidemiology and clinical features of pemphigoid diseases

By far, the most common PD is BP, which predominantly affects patients with a mean age of between 75 and 80 years at the time of diagnosis (18–21). The incidence of BP has recently been prospectively estimated to be 19.6 patients/million/year in Northern Germany (22). About the same incidence has been reported in France, while incidences of 7.63 patients/million/year and 7.1 patients/million/year were described in UK and Sweden based on national health registries (23, 24). In other populations, like in Tehran and Israel, pemphigus is more frequent than BP (25). Annual incidences of the other BP180-related diseases MMP, pemphigoid gestationis, and linear IgA dermatosis were reported to be 1.3 and 2.0/million/year, 0.8–2.0, and 0.25–1.0, respectively (21, 26–29).

BP clinically presents with intense pruritus and tense blisters and erosions. In some patients, urticarial plaques can predominate, and in 20% of patients, no blisters are found (30–32). Pemphigoid gestationis is a mainly transient disease occurring during pregnancy with intense itch and urticarial erythema; vesicles may or may not arise (33). In linear IgA disease, autoantibodies are mainly of the IgA isotype and blistering tends to occur at the edge of lesions, presenting as the so-called crown of jewels sign (34). In contrast to other pemphigoid disorders, in MMP, mucosal surfaces are predominantly involved, manifesting as erosions and crusts, mainly affecting the mouth, conjunctivae, nose, and genital area (26, 35–37).

## Anti-BP230 reactivity in pemphigoid diseases

In 50%–60% of BP patients, in addition to BP180-specific antibodies, reactivity against BP230 is found (38–45). BP patients with autoantibodies restricted to BP230 are rare (40). In pemphigoid gestations, linear IgA diseases, and MMP, anti-BP230 reactivity is less frequent and nearly always accompanied by anti-BP180 antibodies (14, 15, 46). While the main pathogenic effect is mediated by autoantibodies against BP180 (47, 48), the pathogenic effect of anti-BP230 IgG has also recently been described *in vivo* (49).

## Epitopes on BP180

**Figure 1:**
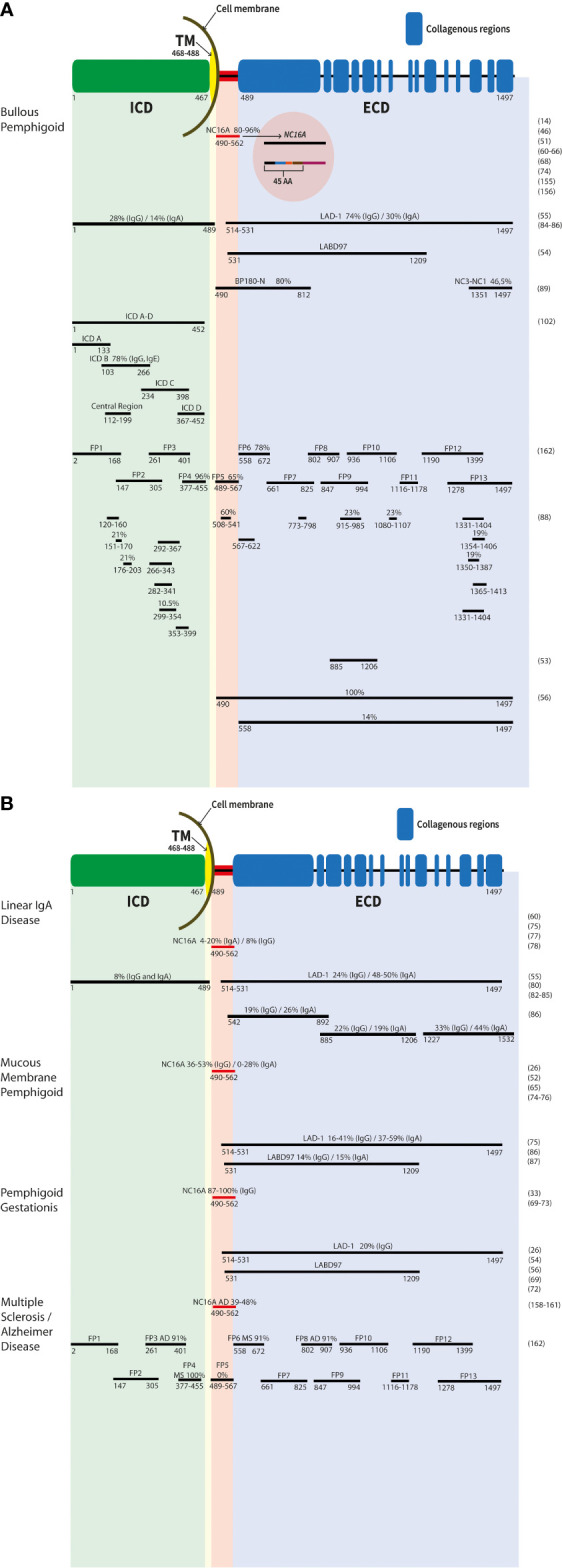
Schematic representation of the antigenic sites of the BP180 protein. The different epitopes of the molecule are shown. Numbers represent the first and last amino acid of each fragment/recombinat protein. The common names are listed above. The main targets are labelled with antibody reactivity (%) and IgG if not otherwise mentioned. Results in bullous pemphigoid **(A)**, linear IgA disease, mucous membrane pemphigoid, pemphigoid gestationis, as well multiple sclerosis and Alzheimer’s disease **(B)** are depicetd. ECD, extracellular domain; ICD, intracellular domain; TM, transmembrane region.

BP180 is a homotrimeric, transmembrane, hemidesmosomal glycoprotein with type II orientation, meaning that the amino-terminal end is located in the cytoplasm while the carboxy-terminus is located in the extracellular space (3, 46) (**Figures 1A, B**). The protein consists of a globular intracellular domain and a large extracellular domain (46, 50). Both domains contain multiple antigenic sites (51–56). The extracellular domain is composed of 15 collagenous domains interrupted by noncollagenous domains called NC1 to NC16 (3, 57, 58). The importance of BP180 for the structural integrity of the DEJ and the attachment of keratinocytes to the underlying dermis is highlighted by mutations in *COL17* encoding for BP180 and resulting in variants of junctional epidermolysis bullosa characterized by blisters and erosions present at birth or in early childhood (59).

In BP, the major antigenic site is the NC16A domain (40). The NC16A domain consists of 76 amino acids and is located immediately outside the transmembrane part (amino acids (aa) 490-562, **Figure 1A**) (46). Interestingly, all reactive sites recognized by autoantibodies are located within the N-terminal region of the NC16A domain and are recognized by IgG autoantibodies in 80%–96% of BP sera (14, 51, 60–65) (**Figure 1A**). For the detection of autoantibodies to BP180, highly sensitive and specific enzyme-linked immunosorbent assays using the NC16A domain as an antigenic target, have been developed recently (60, 61, 64). It has also been shown that disease activity in BP patients correlates with the serum levels of autoantibodies to BP180 NC16A in addition to IgG antibodies, also IgE and IgA autoantibodies are found in BP sera, mainly targeting the NC16A domain (46, 63, 66–68). The NC16A domain of BP180 is also the main target of autoantibodies in pemphigoid gestationis. In total, 87%–100% of cases exhibit IgG autoantibodies against this domain (33, 69–73). Reactivities for IgG and IgA with the NC16A domain were detected in 36%–53% and 0%–28% of MMP patients, respectively (26, 52, 65, 74–76). In LAD, 8% and 4%–20% of analyzed sera exhibit anti-BP180 NC16A IgG or IgA autoantibodies, respectively (75, 77, 78) (**Figure 1B**).

The BP180 ectodomain can be shed within the NC16A domain *via* a disintegrin and metalloprotease (ADAM) 9, 10, and 17 (79–81), resulting in a 120-kDa protein fragment named linear IgA bullous dermatosis autoantigen 1 (LAD-1) and a 97-kDa protein (97-kDa linear IgA bullous dermatosis autoantigen (LABD97)), both originally described as autoantigens in LAD (**Figures 1A,B**) (82–84). By this cleavage within the NC16A domain, different neoepitopes are induced, which might be important for the detection of autoantibodies in LAD, since it was shown that autoantibodies are preferentially bound to LAD-1 than to full-length BP180 (55, 80). By immunoblot analysis, sera from LAD patients showed IgA reactivity against keratinocyte-derived LAD-1 in 48%-50% and IgG reactivity in 24% of cases, whereas sera from BP patients had IgG against LAD-1 in 74% and IgA in 30% of cases (**Figures 1A,B**) (55, 85, 86). In addition, also patients with pemphigoid gestationis and MMP revealed serum reactivity with these two proteins (26, 54, 56, 72). In MMP, 16%–41% and 14% of analyzed sera contained IgG against LAD-1 and LABD97, respectively, and 37%–59% and 15% of sera exhibited IgA reactivity against LAD-1 and LABD97, respectively (**Figure 1B**) (75, 87).

Besides the soluble keratinocyte-derived proteins of the BP180 ectodomain, several recombinant fragments of the BP180 ectodomain have been generated by different investigators (**Figures 1A,B**) (53, 56, 88, 89). In total, 7.8%–47% of BP sera exhibit autoantibodies (IgG and IgA) that are directed against different recombinant proteins of the BP180 ectodomain, excluding the NC16A domain (46, 88–90). IgE against these epitopes has also been detected (91). Recently, in patients without frank blistering but erythematosus lesions, exclusive IgM autoantibodies against BP180 have been reported (92, 93). Boch et al. described three patients who had IgM reactivity against the DEJ by direct IF microscopy and serum IgM against the BP180 ectodomain BMZ. In one patient, additional IgM reactivity against the NC16A domain was present (93). Interestingly, in the last years, it has been reported, that patients with diabetes mellitus treated with dipeptidyl peptidase-4 inhibitors (DPP-4i or gliptins) develop a BP with autoantibodies against epitopes on the C-terminus of BP180 (94–96). In Japanese patients with DPP-4i-associated BP, a noninflammatory phenotype has been described and an association with HLA-DQB1_03:01 was suggested (94, 97).

In MMP, autoantibodies against C-terminal epitopes on BP180 outside NC16A were detected in 16%–53% (IgG) and 4%–37% (IgA) of patients, respectively (26, 46, 52, 74, 75, 98, 99). In pemphigoid gestationis, individual patients exhibit IgG and IgA extracellular epitopes outside NC16A (46, 72). In linear IgA disease, autoantibodies against C-terminal epitopes on BP180 outside NC16A were detected in 50%–100% (46, 55, 77, 80, 100, 101).

Antigenic regions targeted by pemphigoid patients’ autoantibodies can also be found on the intracellular portion of BP180 (88, 102). Overall, 28%–82% of BP sera (IgG reactivity) can recognize at least one epitope of the intracellular domain (56, 88, 102, 103), and also IgE reactivity against this site was described (102). For MMP, serum IgG and IgA against the intracellular part of BP180 were observed in about one-fifth of patients (75). In a small cohort of pemphigoid gestationis patients, about half of the patients showed IgG reactivity with a least one recombinant protein of the intracellular domain of BP180 (72) and in linear IgA disease, IgG and IgA reactivity was detected in 8% (for both isotypes) of analyzed sera (85).

It is currently assumed that intracellular epitopes are only recognized by autoantibodies after there is already reactivity to extracellular domains. Internalization *via* macropinocytosis of a BP180 NC16A IgG immune complex has recently been described (104). Together with the observation that anti-BP230 IgG alone is pathogenic *in vivo* (49, 105), it may be speculated that even autoantibodies against the intracellular part of BP180 per se are pathogenic. Of note, the autoantibody profile in individual PD patients is dynamic and may change over time, a phenomenon called epitope spreading. So far, little is known about the pathogenic relevance of autoantibodies against the intracellular and non-NC16A epitopes on the BP180 ectodomain. Alike, clinical phenotypes within a distinct PD depending on the targeted epitopes have not yet been sufficiently explored. Setterfield et al., however, have found concomitant serum IgG and IgA reactivity against the DEJ to be associated with a more severe phenotype in MMP (106).

## Fcγ-receptor-independent effects of BP180-specific antibodies

Tissue destruction in BP is mediated by a variety of Fcγ-receptor (FcγR)-mediated effects. The antigen-autoantibody complexes lead to the activation of complement, followed by the infiltration of inflammatory cells such as mast cells, macrophages, and neutrophils to the upper dermis (107–110). These infiltrating cells release reactive oxygen species and specific proteases which finally degrade the DEJ, clinically leading to blister formation (109, 111). Most data about the essential role of complement activation in BP stems from observations in the neonatal mouse model of this disease (107, 108, 112). These data were supported by the high impact of complement activation in the mouse models of EBA and MMP (113–115).

Of note, antibodies against BP180 also promote blister formation in a FcγR- and complement-independent manner (116). The binding of anti-BP180 antibodies led to reduced adhesion of keratinocytes to the substrate/basement membrane using cultures of human keratinocytes and skin by macropinocytosis/internalization (104, 117–119). In detail, it was shown that BP-IgG induced a 45%–50% depletion of BP180 from normal human keratinocytes and DJM-1 cells (malignant trichilemmal cyst cells) after 2 h of incubation and about 75% in DJM-1 cells and 85% in normal human keratinocytes after 6 h. BP patient’s sera had the same depletion ability. A depletion of 80%–95% of BP180 from cells compared to normal control sera could be observed in seven of nine sera, the remaining two had a depletion activity of about 50%. The depletion of BP180 led to a loss of adhesion strength of about 40% in normal human keratinocytes and DJM-1 cells (119).

The internalization of BP180 upon binding of anti-BP180 IgE to cultured keratinocytes was also observed by confocal microscopy. After a time period of 15 min, the anti-BP180 IgE staining was detectable on the cell surface and 45 min later, the staining had moved from the periphery to the center, i.e., the cytoplasm of the cultured keratinocytes (117). The authors suggested that the possible blistering-inducing potential of anti-BP180 IgE is derived from its more potent cytokine-inducing potential compared to anti-BP180 IgG. This view was based on the observation that the relation of serum anti-BP180 IgE to total serum IgE was 30%–40% considerably higher compared to less than 1% of anti-BP180 IgG in relation to total serum IgG (117). These data are in line with the results Hiroyasu et al. observed for the internalization of BP IgG (118). They used BP-IgG to treat subconfluent/confluent normal human keratinocyte cultures that expressed green fluorescent protein-BP180 (GFP-BP180) and observed the beginning of an internalization, described as spot-like structures, from the surface into the cytoplasm after 20/105 min. By additionally labeling the BP-IgG with Fluor 647, it turned out that BP180 is internalized together with its antibody no matter if the antibodies are bound to the intracellular domain or extracellular domain (104). Internalization occurred *via* a non-inflammatory pathway and is FcγR-independent. This was tested using BP-IgG Fab fragments for cell stimulation, where an internalization of GFP-BP180 still occurred. By additionally treating the cells with EIPA, a Na+/H+ exchanger inhibitor, and cytochalasin D, an actin polymerization inhibitor, the internalization of GFP-BP180 failed. This indicates that internalization occurs *via* a macropinocytotic pathway (118). This process was suggested to be mediated by the protein kinase C pathway *via* phosphorylation of BP180 followed by the internalization and degradation of immune complexes, leading to a deficiency of BP180 in cell membranes and, consequently, loss of adhesion strength in keratinocytes (104). Furthermore, Kamaguchi and co-workers showed for MMP that the interaction between collagen type IV and BP180 is disrupted by MMP IgG directed against the BP180 C-terminus in keratinocytes of the oral mucosa, resulting in a reduction of the adhesion to the BMZ. However, the mechanism of lesion formation in the mucosa is still not fully understood. It has been shown that the expression level of BP180 is higher in the oral mucosa compared to the skin (120). This in turn is thought to compensate for the depletion of BP180 induced by anti-BP180 NC16A IgG.

The importance of the release of inflammatory cytokines like IL-6 and IL-8 in the pathogenesis of BP is supported by the finding of increased levels of both cytokines in the blister fluid of BP patients compared to suction blister controls (111, 121). Furthermore, a correlation between IL-6 and IL-8 serum levels and BP disease activity was shown (122). The release of IL-6 and IL-8 upon stimulation of cultured human keratinocytes with BP patient IgG indicates that FcγR-independent events might be relevant for disease development. Schmidt et al. observed a dose- and time-dependent release of the two cytokines. The elevated IL-6 and IL-8 levels in this approach were shown to be mediated by anti-BP180 IgG and were also observed at the mRNA level (123). Subsequently, the IL-8 release was shown to be significantly inhibited by dapsone but not doxycycline (124), two drugs regularly used in the treatment of BP (125–127). These data were corroborated by the observation that the treatment of cultured keratinocytes with IgE against BP180 also leads to the release of IL-6 and IL-8 (117). These cytokines may directly attract neutrophils without complement activation, leading to blister formation (128).

In addition, the injection of anti-BP180 IgG into humanized neonatal C3-deficient mice resulted in skin detachment by depletion and degradation of BP180 without complement deposits (107). This was somehow unexpected since, in C5-deficient mice, no dermal–epidermal separation occurred (107). Supporting the findings in C3-deficient animals, injection of F(ab′)2 fragments resulted in skin fragility without complement activation and neither skin detachment nor complement deposits in C5-deficient mice (107). Collectively, these data support the hypothesis that both FcγR- and complement-independent processes are involved in the pathophysiology of BP.

## Anti-BP180 reactivity in neurological disease with and without concomitant bullous pemphigoid

BP is often associated with neurological diseases such as Alzheimer’s disease, Parkinson’s disease, and multiple sclerosis (129–133). Proteins associated with neurological diseases, like amyloidogenic proteins, were detected in human skin (134). In Alzheimer’s disease patients, skin physiology was altered and their risk of developing BP was 2.6 times higher than in individuals without the neurological disease (134–136). On the other hand, patients with nonmelanoma skin cancer had a lower risk of developing Alzheimer’s disease (137). One explanation for both observations is that the main antigen for BP, BP180, is expressed not only in the skin but also in the central nervous system (CNS) (138, 139). It has further been suggested that due to the neurodegenerative processes in neurological diseases the immune privilege of the CNS may be lost, i.e., that the blood–brain barrier no longer protects against harmful substances, pathogens, and toxins (140). This loss can then lead to the accessibility of the antigens in the brain and a subsequent autoimmune response against BP180 and BP230 (129, 133). More precisely, Seppänen et al. localized BP180 in the soma and proximal axons, but not in glial cells (139). BP180 was mostly found in the pyramidal cells of hippocampal regions, the ganglionic layer of the cerebral cortex, the hypoglossal nucleus (nucleus XII), oculomotor nucleus (nucleus III), nucleus basalis of Meynert, supraoptic nucleus, and subthalamic nuclei (139, 141). The observation that the neurological disease precedes BP by an average of 5.5 years supports the hypothesis that, indeed, the neurological disease triggers the autoimmune blistering disorder (130, 142). In fact, the relationship between BP and stroke, dementia, Parkinson’s disease, multiple sclerosis, and epilepsy has been well established in various populations (129–131, 133, 142–153). Since both neurological diseases such as stroke or dementia increase in the elderly and an age-related impairment of the blood-brain barrier has been described, this may also explain the striking occurrence of BP above the age of 70 years (140, 154–158).

To provide further experimental evidence for the generation of anti-BP180 IgG in patients with neurological diseases, Kokkonen and co-workers tested 115 Alzheimer’s disease patients and 40 neurologically healthy controls for anti-BP180 IgG. 21% of the patients with Alzheimer’s disease but only 7.5% of the controls showed IgG against BP180 NC16A by ELISA (159). All positive samples were further analyzed by immunoblotting against recombinant full-length BP180. In total, 18% of Alzheimer’s disease patients revealed IgG against NC16A and full-length BP180 in contrast to 3% in controls. Interestingly, Alzheimer’s disease patients with higher serum anti-BP180 IgG levels had more severe dementia (159). Comparable results were obtained by Wang et al., who found NC16A ELISA reactivity in 48% of 23 Alzheimer’s disease patients (160) (**Figure 1B**). In the sex- and age-matched healthy control group, only 8% of the tested 50 sera reacted with NC16A. Validation by immunoblot analyses with recombinant full-length BP180 or NC16A showed that nine of the 23 ELISA-positive Alzheimer’s disease patients (39%) and one of four (25%) controls reacted with the proteins (**Figure 1B**). In addition, 11 of the 23 ELISA-positive Alzheimer’s disease sera reacted with a 180-kDa protein from the human brain extract, but none of the controls did. The brain extract was obtained from the human hippocampus, which is a known BP180 expression locus (160). In contrast, Recke et al. found no significant increase in autoreactivity of autoantibodies against BP180 in patients with multiple sclerosis and Parkinson’s disease (161). The latter study is compatible with further work by Tuusa et al. that detected serum anti-BP180 NC16A IgG in only eight of 143 (5.6%) patients with multiple sclerosis and two of 140 (1.4%) neurologically healthy controls by ELISA, while none of these sera reacted with a glutathione-*S*-transferase NC16A fusion protein by immunoblotting (162). When different fusion proteins (FP1-FP13) covering BP180 (**Figures 1A,B**) were employed, BP sera preferentially reacted with the extracellular fragment FP5, corresponding to the NC16A domain, while there was no reactivity in sera of patients with multiple sclerosis, Alzheimer’s disease and healthy controls. Of note, patients with multiple sclerosis primarily reacted with the intracellular fragment FP4 (in 100%) and the extracellular fragments FP6 outside the NC16A domain (in 91%). Alzheimer’s disease sera predominantly recognized the intracellular fragment FP3 (in 91%) and the extracellular fragments FP8 outside the NC16A domain (in 91%; **Figure 1**) (162). Collectively, these data indicate that autoantibodies against different epitopes in BP180 are associated with different pathologies and as such, it can be expected that the pathogenic effect of anti-BP180 autoantibodies not only depends on their complement-activating potential, their glycosylation status, and isotype but also on the targeted epitope.

## Outlook and conclusions

It has been well documented that different epitopes on BP180 are targeted in different PD as well as in patients with neurodegenerative disorders. The main pathogenic effect of anti-BP180 autoantibodies appears to be mediated by FcγR. Correspondingly, the pathogenicity of anti-BP180 autoantibodies was shown to depend on the autoantibody isotype, IgG subclass, and glycosylation status, leading to a varying extent of complement activation at the DEJ and attraction of inflammatory cells to the upper dermis. A more puzzling observation was that in different PDs, different regions of BP180 are predominantly targeted. In addition, most *in vitro* and all animal models have shown pathogenicity only against the NC16A domain and its murine homolog, not against intracellular or extracellular epitopes outside of this domain. In line, only serum levels of antibodies against the NC16A domain were shown to correlate with the disease activity of patients. As such, different epitopes on BP180 may convey different pathologies. This hypothesis, however, cannot be well explained by FcγR-dependent mechanisms alone.

Increasing evidence is provided for FcγR-independent mechanisms of anti-BP180 IgG in different *in vitro* and *in vivo* models. These mechanisms would allow us to more closely investigate the role of different epitopes in the pathophysiology of PD. The targeted epitope(s) may vary among the different disease stages and may also play a role in the transition of pemphigoid predisease, i.e., when autoantibodies against the DEJ are present but no skin or mucosal lesions have yet evolved. The in-depth characterization of epitope-dependent disease mechanisms in PD will not only allow a better understanding of autoantibody-triggered inflammation but may also provide novel therapeutic approaches that are urgently needed.

## Author contributions

BO, ES, and SG contributed to the writing of the manuscript. ES contributed to the revision of the manuscript. All authors contributed to the article and approved the submitted version.

## Funding

RTG 2633, Autoimmune Pre-Disease, University of Lübeck, Spokesperson: Ralf Ludwig.

## Conflict of interest

The authors declare that the research was conducted in the absence of any commercial or financial relationships that could be construed as a potential conflict of interest.

## Publisher’s note

All claims expressed in this article are solely those of the authors and do not necessarily represent those of their affiliated organizations, or those of the publisher, the editors and the reviewers. Any product that may be evaluated in this article, or claim that may be made by its manufacturer, is not guaranteed or endorsed by the publisher.
